# SNORA74B gene silencing inhibits gallbladder cancer cells by inducing PHLPP and suppressing Akt/mTOR signaling

**DOI:** 10.18632/oncotarget.15301

**Published:** 2017-02-13

**Authors:** Yiyu Qin, Li Meng, Yang Fu, Zhiwei Quan, Mingzhe Ma, Mingzhe Weng, Zhengdong Zhang, Cuixiang Gao, Xinghua Shi, Koulan Han

**Affiliations:** ^1^ Clinical College, Yancheng Institute of Health Sciences, Yancheng, Jiangsu 224000, China; ^2^ Research Centre of Biomedical Technology, Yancheng Institute of Health Sciences, Yancheng, Jiangsu 224000, China; ^3^ Department of General Surgery, Xinhua Hospital Affiliated to Shanghai Jiao Tong University School of Medicine, Shanghai 200092, China; ^4^ Department of Environmental Genomics, Jiangsu Key Laboratory of Cancer Biomarkers, Prevention and Treatment, Collaborative Innovation Center For Cancer Personalized Medicine, Nanjing Medical University, Nanjing 210029, China; ^5^ Department of Gastrointestinal Surgery, The First Affiliated Hospital of Zhengzhou University, Zhengzhou, Henan 450052, China

**Keywords:** gallbladder cancer, SNORA74B, PHLPP, snoRNA, AKT

## Abstract

Small nucleolar RNAs (snoRNAs) have been implicated in the development of many cancers. We therefore examined the differential expression of snoRNAs between gallbladder cancer (GBC) tissues and matched adjacent non-tumor tissues using expression microarray analysis with confirmation by quantitative real-time PCR (qRT-PCR). Western blot analysis showed that SNORA74B levels were higher in GBC than non-tumor tissues. SNORA74B expression was positively associated with local invasion, advanced TNM stage, CA19-9 level, and Ki67 expression in patients with GBC, while it was negatively associated with expression of PHLPP, an endogenous Akt inhibitor. Moreover, SNORA74B expression was prognostic for overall survival (OS) and disease-free survival (DFS). Functional studies revealed that silencing SNORA74B in GBC cells using sh-SNORA74B suppressed cell proliferation, induced G1 arrest, and promoted apoptosis. Preliminary molecular investigation revealed that SNORA74B silencing inhibited activation of the AKT/mTOR signaling pathway, while increasing PHLPP expression. PHLPP depletion using shRNA abrogated sh-SNORA74B suppression of GBC cell proliferation, indicating that the antitumor effects of SNORA74B silencing were mediated by PHLPP. These findings define the important role of SNORA74B in cell proliferation, cell cycle, and apoptosis of GBC, and suggest that it may serve as a novel target for GBC treatment.

## INTRODUCTION

GBC is the most common malignancy of the biliary tract and the fifth most common malignancy of the gastrointestinal tract worldwide [1]. Due to vague symptoms in the early stage, most GBC cases are diagnosed at an advanced stage when nodal involvement, hepatic invasion, and metastatic progression are present, greatly increasing the difficulty of treatment. Despite progress in GBC treatment over the last few decades, the prognosis of GBC remains extremely poor with an overall mean survival of 6 months and 5-year survival rate of less than 5% [2]. Because the etiology and pathogenesis of GBC are unknown, it is imperative to study the molecular mechanisms of GBC pathogenesis in order to find novel cancer biomarkers that may help obtain early diagnosis and therapy.

Small nucleolar RNAs (snoRNAs) are a class of non-coding regulatory RNAs about 60-300 nucleotides in length mainly located in the cell nucleolus. While it was originally believed that snoRNAs are primarily engaged in the processing of rRNA, recent studies have revealed many unexpected cellular functions for snoRNAs which may change entrenched views of gene expression. During the last decade, hundreds of snoRNAs throughout the human body have been linked to miRNA genesis, alternate splicing, and stress response [3]. Since snoRNAs play an important role in many physiological conditions, the alteration in expression of snoRNAs may lead to various diseases. The relationship between snoRNAs and tumors has been documented in recent studies. It has been reported that SNORA42 is frequently overexpressed in non-small-cell-lung carcinoma (NSCLS) and colorectal cancer, and that siRNA knockdown of SNORA42 results in reduced cancer cell growth, indicating that SNORA42 is a putative oncogene [4, 5]. On the other hand, the tumor suppressive role of the SNORD113-1 gene has been confirmed in hepatocellular carcinoma (HCC). SNORD113-1 expression is abnormally downregulated in HCC tissues compared with adjacent non-tumor tissues, and it may suppress growth of HCC cell both *in vitro* and *in vivo* [6], demonstrating that snoRNAs may have a dual role in tumor development.

In the present study, we examined the differential expression of a set of snoRNAs between tumor and non-tumor tissue with the hope of finding snoRNAs directly linked to cancer progression that may serve as new diagnostic and prognostic markers.

## RESULTS

### SNORA74B expression profile in GBC tissues and cell lines

**Figure 1:**
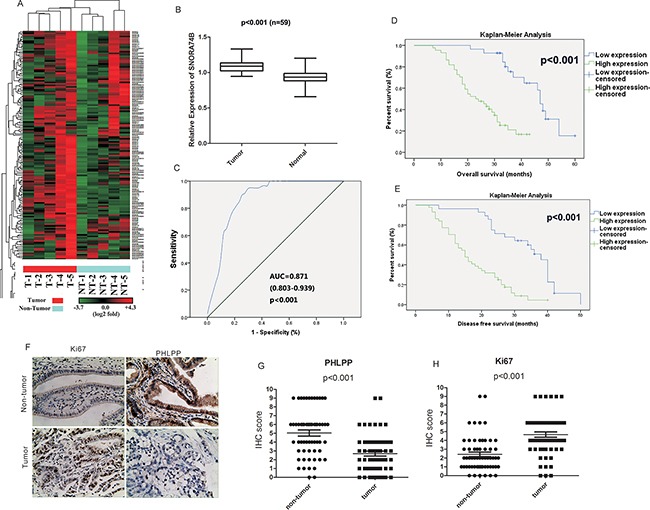
SNORA74B expression profile in GBC tissues and cell lines **A**. Hierarchical clustering analysis of the snoRNAs that were differentially expressed (p < 0.05) between GBC samples (T, tumor) and matched non-tumor samples (NT, non-tumor). **B**. SNORA74B expression was measured by qRT-PCR in GBC tissues and adjacent non-tumor tissues (n = 59). SNORA74B expression level was normalized to that of U6. **C**. Identification of SNORA74B as a gene biomarker for GBC by ROC curve analysis. The area under curve (AUC), 95% CI, and *p* value are shown. Correlation between overall survival (OS) **D**. and disease-free survival (DFS) **E**. and SNORA74B expression of GBC patients by Kaplan-Meier survival analysis. **F**. Immunohistochemistry of Ki67 and PHLPP in GBC patients (×200). Immunostaining scores of PHLPP **G**. and Ki67 **H**. in GBC patients. Normal: non-tumor.

Microarray analysis was performed to identify differentially expressed transcripts involved in GBC tumorigenesis. A hierarchical clustering analysis showed systematic variations in snoRNA expression levels between GBC tissues and matched adjacent nontumor tissues from 5 GBC patients (Figure 1A). The microarray result shows that 115 snoRNAs are differentially expressed. Among these snoRNAs, 74 is up-regulated and 41 are down-regulated. In addition, 16 snoRNAs have at least 2-fold up-regulation and 5 snoRNAs with 2-fold down-regulation. Detailed data are listed in Table 1. qRT-PCR results for these 21 snoRNAs with FC > 2 or FC < 0.5 indicates SNORA74B, SNORA21, SNORD71A, SNORD38b, SNORD20 and SNORD75 have the highest up-regulation in GBC tissue. These data are also listed in Table 1.

**Table 1:** Differetially expressed snoRNAs with microarray and qRT-PCR

Gene symbol	snoRNA array			qRT-PCR	
	p value	Fold Change (FC)	FDRs	p value	Fold Change (FC)
SNORA76	0.008	4.038	0.373	0.023	2.343
SNORA74B	0.022	3.952	0.442	<0.001	4.568
SNORA21	<0.001	3.927	0.482	<0.001	3.732
SNORD20	0.022	3.435	0.128	0.005	3.545
SNORA33	0.047	2.908	0.442	0.003	2.534
SNORD38b	0.002	2.892	0.321	0.002	3.413
SNORA71A	<0.001	2.784	0.468	<0.001	3.232
SNORA64	0.022	2.619	0.442	0.031	1.134
SNORD12	0.001	2.49	0.415	0.007	1.598
SNORA14B	0.04	2.434	0.387	0.004	1.66
SNORD59A	0.017	2.399	0.549	0.029	2.458
SNORA46	0.014	2.306	0.321	0.018	2.367
SNORA75	0.003	2.228	0.321	0.003	2.959
SNORA71C	0.003	2.2	0.481	0.007	2.035
SNORD83A	0.049	2.158	0.567	0.021	1.994
SNORA9	0.035	2.008	0.455	0.035	2.751
ENST00000364166	0.006	0.468	0.373	0.009	1.589
SNORD114-2	0.003	0.45	0.321	0.015	2.43
SNORD115-44	0.048	0.444	0.482	0.027	1.435
SNORD116-21	0.018	0.418	0.415	0.023	0.322
ENST00000363156	0.037	0.377	0.455	0.007	0.676

**Figure 2:**
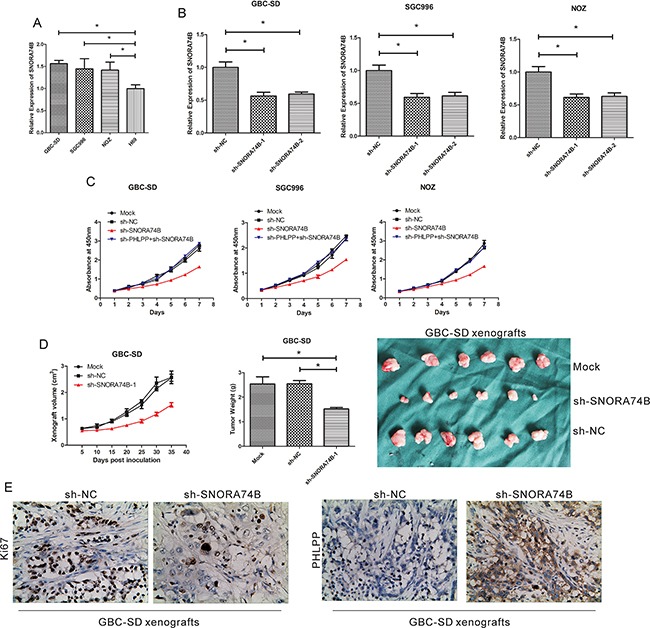
SNORA74B silencing inhibits GBC cell proliferation **A**. SNORA74B expression profiles in tumor cell lines (GBC-SD, SGC996, and NOZ) and non-tumor cell line H69. **B**. Silencing of SNORA74B gene in GBC cell lines by shRNAs. Mock: cells treated with no shRNA. sh-NC: cells treated with sh- scrambled. **C**. Silencing of SNORA74B gene inhibits proliferation of GBC-SD, SGC996, and NOZ cells *in vitro*. Effects of SNORA74B knockdown on xenograft volume and weight *in vivo*. GBC-SD xenografts were shown after treatment with shRNAs **D**. **E**. IHC staining of Ki67 and PHLPP in xenografts formed by SNORA74B-silenced GBC-SD cells (×200).

The expression levels analysis in 59 GBC tissues and matched non-tumor tissues (Figure 1B) indicated that SNORA74B expression is aberrantly up-regulated in tumor tissues (p<0.001). In addition, we examined SNORA74B expression in GBC-SD, SGC996, NOZ and H69 cell lines. As shown in Figure 2A, GBC-SD, SGC996 and NOZ cells display aberrantly overexpression of SNORA74B, while the level of SNORA74B expression in H69 is much lower than in cancer cell lines. Moreover, to determine the sensitivity and specificity of SNORA74B expression to discriminate tumor tissues from non-tumor tissues, receiver operating characteristic (ROC) curve analysis was performed. SNORA74B was proven to be a predictor with considerable clinical significance, with an area under curve(AUC) of 0.871 (95% CI (confidence interval) = 0.803–0.939, p<0.001; Figure 1C).

### Prognostic and clinicopathological features of SNORA74B in GBC

Next, to determine the clinical significance of SNORA74B expression for GBC patients, we analyzed the association between SNORA74B expression and clinicopathological characteristics. The patients were divided into a low SNORA74B expression group (n=28) and a high SNORA74B expression group (n=44) according to the mean value of relative SNORA74B expression. The detailed correlation between SNORA74B expression levels and clinicopathological characteristics of GBC patients are shown in Table 2. A higher SNORA74B expression level was positively associated with increased local invasion (p=0.008), advanced AJCC tumor stage (p=0.011), increased carbohydrate antigen 19-9 (CA 19-9, p=0.041), and high expression of Ki67 (p=0.021), while it was negatively associated with expression of PHLPP (p=0.002). The immunohistochemical staining (Figure 1F, 1G, 1H) revealed that Ki67 protein was significantly increased, while PHLPP protein level was downregulated in GBC tissues. Kaplan-Meier analysis suggested a correlation between high tumor SNORA74B expression and reduced overall survival (OS) and disease-free survival (DFS) rates (p< 0.05 for both OS and DFS, Figure 1D, 1E). Furthermore, univariate analysis identified the expression of SNORA74B as well as local invasion, lymph-node metastasis, distant metastasis, TNM staging, CA19-9 level, Ki67 expression as negative prognosticators for GBC, while PHLPP may serve as a good prognosticator (P < 0.05) (Table 3). The final multivariate analysis indicated that SNORA74B overexpression in GBC was an independent predictor of shorter survival (HR = 3.309, CI = 1.257-8.709, p = 0.015) (Table 4).

**Table 2:** The relationship of SNORA74B expression and clinicopathological characteristics in GBC

	Low expression of SNORA74B n (%)	High expression of SNORA74B n (%)	p value
Age (years)			
<60	11	20	
≥60	17	24	0.606
Gender			
Male	8	17	
Female	20	27	0.382
Local invasion			
T1	9	5	
T2	10	7	
T3	6	18	
T4	3	14	0.008*
Lymph node metastasis			
Yes	10	16	
No	18	28	0.955
Distant metastasis			
Yes	11	11	
No	17	33	0.2
TNM stage			
I-II	16	12	
III-IV	12	32	0.011*
CA19-9 (U/ml)			
<74	15	13	
≥74	13	31	0.041*
CEA (μg/L)			
<11.8	14	19	
≥11.8	14	25	0.571
CA125 (U/ml)			
<35	12	15	
≥35	16	29	0.454
Ki67			
Low	18	16	
High	10	28	0.021*
PHLPP			
Low	8	29	
High	20	15	0.002*

**Table 3:** Univariate analysis for prognostic factors of GBC

Variables	group	case	average survival time(months) 95% CI	P value
Gender	male	25	31.992(28.070-35.915)	0.244
	female	47	36.893(29.527-44.260)	
Age(years)	<60	31	33.157(28.068-38.246)	0.884
	≥60	41	34.246(29.041-39.451)	
Local invasion	T1	14	44.333(36.666-52.001)	0.001*
	T2	17	33.613(29.242-37.983)	
	T3	24	32.276(26.561-37.990)	
	T4	17	22.588(17.555-27.622)	
Lymph node metastasis	Yes	26	27.796(22.266-33.327)	0.034*
	No	46	37.285(32.559-42.012)	
Distant metastasis	Yes	22	24.992(19.835-30.149)	0.004*
	No	50	37.641(33.162-42.119)	
TNM stage	I-II	28	42.969(37.479-48.459)	0.001*
	III-IV	44	28.252(24.189-32.316)	
CA19-9 (U/ml)	<74	28	41.024(35.735-46.314)	0.006*
	≥74	44	28.781(24.482-33.079)	
CEA (μg/L)	<11.8	33	38.319(32.311-44.327)	0.051
	≥11.8	39	30.352(25.852-34.851)	
CA125 (U/ml)	<35	27	32.428(26.715-38.141)	0.322
	≥35	45	35.654(30.459-40.849)	
Ki67	Low	34	41.658(35.719-47.597)	0.013*
	High	38	29.600(25.485-33.716)	
PHLPP	Low	37	24.804(21.204-28.403)	<0.0001*
	High	35	45.042(40.046-50.037)	
SNORA74B	Low	28	45.423(40.900-49.946)	<0.0001*
	High	44	25.246(21.872-28.621)	

**Table 4:** Multivariate analysis for prognostic factors of GBC

Variables	HR	95%CI	p value
SNORA74B	3.309	1.257-8.709	0.015*
local invasion			0.303
local invasion (1)	1.061	0.29-8.882	0.929
local invasion (2)	0.35	0.32-3.809	0.389
local invasion (3)	0.752	0.66-8.634	0.819
lymph node metastasis	2.254	0.533-2.947	0.063
distant metastasis	1.883	0.402-1.941	0.157
TNM	3.236	0.403-25.958	0.026*
CA199	3.762	0.531-26.642	0.185
Ki67	0.288	0.039-2.11	0.221
PHLPP	0.329	0.137-0.790	0.013*

### SNORA74B silencing inhibited GBC cell proliferation

Because SNORA74B expression is up-regulated in GBC-SD, SGC996, and NOZ cells, we used shRNA to silence SNORA74B expression for functional studies. Two specific shRNAs for SNORA74B (sh-SNORA74B-1 and sh-SNORA74B-2) were transfected into GBC-SD, SGC996, and NOZ cells (Figure 2B); both effectively suppressed the expression of SNORA74B (p < 0.001). sh-SNORA74B-1 was used for further functional studies. CCK-8 assay results showed that sh-SNORA74B suppressed proliferation of GBC-SD, SGC996, and NOZ cells significantly (Figure 2C). Moreover, the volume of xenografts formed by SNORA74B-silencing cells was significantly smaller than those formed by control cells, and the same trends were observed in weight of xenografts between SNORA74B-silencing and control groups (Figure 2D). In addition, the expression of Ki67 protein was significantly decreased in xenografts formed by SNORA74B-silenced cells (Figure 2E). These data indicated that SNORA74B silencing inhibits tumor growth both *in vitro* and *in vivo*.

### SNORA74B silencing induced G1 arrest of GBC cells

**Figure 3:**
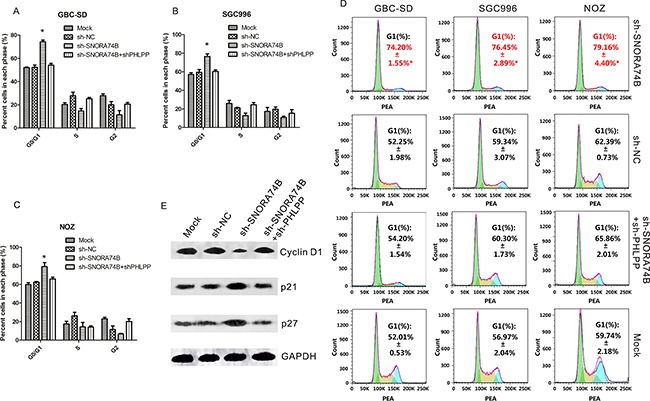
SNORA74B silencing induced G1 arrest of GBC cells **A-C**. The relative cell numbers in G0/G1, S, and G2 phase after propidium iodide staining of SNORA74B-silenced cells were determined by FACS. **D**. FACS data was analyzed using FlowJo v7.6.2 (Tree Star, Ashland, OR, USA). Data are presented as mean ± S.D. **E**. Expression of G1 checkpoint regulators p21, p27, and cyclin D1 were examined by western blotting, and the values of relative density of the bands normalized to GAPDH are shown.

As shown in Figure 3A-3D, the G1 population of GBC-SD (74.20%±1.55%*), SGC996 (76.45%±2.89%*) and NOZ (79.16%±4.40%*) cells was significantly increased after transfection with sh-SNORA74B compared to control groups (p<0.05), and a corresponding significant reduction of S + G2 population of cells was observed. In addition, the levels of G1 checkpoint regulators such as p21, p27, and cyclin D1 were examined by western blotting. sh-SNORA74B induced p21 and p27 protein expression, while it significantly inhibited the expression of cyclin D1 protein (Figure 3E). The relative gray values of each band normalized to GAPDH or COX IV were shown in Table 5.

**Table 5:** Relative gray value of protein bands after treatments in Figure 3E, 4E, 5A, 5B and 5C

Figure 3E					
Protein	Mock	sh-NC	sh-SNORA74B	sh-SNORA74B+sh-PHLPP	
cyclin D1	1	1.16	0.35	1.04	
	1	1.06	0.46	0.96	
	1	1.02	0.42	0.94	
	1	1.08	0.41	0.98	mean
	0	0.072	0.056	0.053	SD
p21	1	1.15	2.06	1.09	
	1	0.94	1.95	1.02	
	1	1.06	1.93	1.07	
	1	1.05	1.98	1.06	mean
	0	0.105	0.07	0.036	SD
p27	1	1.15	2.2	1.01	
	1	0.99	2.11	0.93	
	1	1.22	2.14	0.94	
	1	1.12	2.15	0.96	mean
	0	0.118	0.046	0.044	SD
GAPDH	1	1	1	1	
Figure **4E**					
bcl-2	1	0.97	0.35	0.91	
	1	0.92	0.36	0.91	
	1	1.02	0.4	0.94	
	1	0.97	0.37	0.92	mean
	0	0.05	0.026	0.017	SD
bax	1	0.85	1.86	0.96	
	1	0.94	1.88	1.02	
	1	1.06	1.93	0.99	
	1	0.95	1.89	0.99	mean
	0	0.105	0.036	0.03	SD
procapase-3	1	0.93	0.98	1.05	
	1	0.99	0.99	1.01	
	1	0.93	0.79	0.94	
	1	0.95	0.92	1	mean
	0	0.034641	0.1126943	0.0556776	SD
cleaved caspase-3	1	0.89	1.98	0.91	
	1	1.05	2.11	1.03	
	1	0.88	2	0.73	
	1	0.94	2.03	0.89	mean
	0	0.095	0.07	0.151	SD
GAPDH	1	1	1	1	
mitochondrial cytochrome C	1	0.99	0.41	1.05	
	1	0.99	0.42	1.09	
	1	1.26	0.46	1.16	
	1	1.08	0.43	1.1	mean
	0	0.156	0.026	0.056	SD
cytosolic cytochrome C	1	0.99	2.08	1.05	
	1	1.03	2.15	1.03	
	1	1.16	2.19	1.04	
	1	1.06	2.14	1.04	mean
	0	0.089	0.056	0.01	SD
COXIV	1	1	1	1	
Figure **5A**					
AKT	1	1.03	1.09	1.06	
	1	1.07	1.08	1.12	
	1	1.02	0.98	1.06	
	1	1.04	1.05	1.08	mean
	0	0.026	0.061	0.035	SD
p-AKT	1	0.8	0.29	0.96	
	1	1.05	0.4	1.02	
	1	1.06	0.36	1.08	
	1	0.97	0.35	1.02	mean
	0	0.1473092	0.0556776	0.06	SD
Mtor	1	0.93	0.98	1.05	
	1	0.99	0.99	1.01	
	1	1.14	1.06	0.85	
	1	1.02	1.01	0.97	mean
	0	0.108	0.044	0.106	SD
p-Mtor	1	0.99	0.19	0.98	
	1	1.05	0.28	1.03	
	1	1.2	0.34	1.14	
	1	1.09	0.27	1.05	mean
	0	0.108	0.0753	0.082	SD
4EBP1	1	1.03	1.01	0.99	
	1	1.05	1.09	1.03	
	1	1.13	1.14	1.04	
	1	1.07	1.08	1.02	mean
	0	0.053	0.066	0.026	SD
p-4EBP1	1	0.98	0.34	0.89	
	1	1	0.35	1.02	
	1	1.05	0.39	1.03	
	1	1.01	0.36	0.98	mean
	0	0.036	0.026	0.078	SD
p70 S6K	1	0.93	0.91	0.98	
	1	0.95	0.94	1.01	
	1	1.12	1.03	1.13	
	1	1	0.96	1.04	mean
	0	0.104	0.062	0.079	SD
p-p70 S6K	1	0.96	0.42	1.01	
	1	0.95	0.41	1.03	
	1	1.15	0.52	1.11	
	1	1.02	0.45	1.05	mean
	0	0.113	0.061	0.053	SD
bad	1	0.86	0.98	1.02	
	1	0.85	1.01	0.93	
	1	1.05	1.07	0.87	
	1	0.92	1.02	0.95	mean
		0.113	0.046	0.075	SD
p-bad	1	0.9	0.45	0.96	
	1	0.96	0.47	1.03	
	1	0.96	0.52	1.04	
	1	0.94	0.48	1.01	mean
	0	0.035	0.036	0.044	SD
GAPDH	1	1	1	1	
Figure **5B**					
PTEN	1	0.95	0.92	0.91	
	1	0.96	0.94	0.92	
	1	1	1.02	0.96	
	1	0.97	0.96	0.93	mean
	0	0.026	0.053	0.026	SD
CTMP	1	0.97	1.06	1.01	
	1	0.97	1.05	1.03	
	1	1.09	1.13	1.14	
	1	1.01	1.08	1.06	mean
	0	0.069	0.044	0.07	SD
PHLPP	1	1.01	2.51	0.96	
	1	1.05	2.68	0.99	
	1	1.09	2.7	0.99	
	1	1.05	2.63	0.98	mean
	0	0.04	0.104	0.017	SD
GAPDH	1	1	1	1	
Figure **5C**					
PHLPP	1	0.2		0.09	
	1	0.24		0.1	
	1	0.25		0.14	
	1	0.23		0.11	mean
	0	0.026		0.0265	SD
PHLPP	1	0.18		0.06	
	1	0.19		0.09	
	1	0.2		0.09	
	1	0.19		0.08	mean
	0	0.01		0.017	SD
PHLPP	1	0.15		0.08	
	1	0.18		0.08	
	1	0.18		1.11	
	1	0.17		0.09	mean
	0	0.017		0.595	SD
GAPDH	1	1		1	

### SNORA74B silencing promoted GBC cell apoptosis

**Figure 4:**
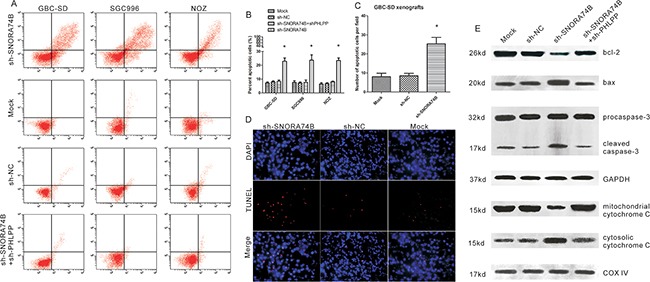
SNORA74B silencing promoted GBC cell apoptosis **A**. FACS determined the relative number of apoptotic cells after double staining with Annexin V and PI. X axis represents the level of Annexin-V FITC fluorescence and the Y axis the PI fluorescence. **B**. The percent of apoptotic cells for GBC-SD, SGC996, and NOZ (p<0.05). **C**. The number of TUNEL-positive cells for GBC-SD xenografts. **D**. Effects of SNORA74B silencing on apoptosis *in vivo* were detected using TUNEL assay. The blue fluorescence indicates nuclear staining (DAPI) and the red fluorescence represents TUNEL-positive nuclei of GBC-SD cells. **E**. Apoptosis regulators were examined using western blotting, and the values of relative density of the bands normalized to GAPDH and COX IV are shown.

Induction of apoptosis by SNORA74B silencing *in vitro* and *in vivo* was determined using Annexin V/PI assay and TUNEL assay, respectively. As shown in Figure 4A and 4B, the population of apoptotic cells was significantly increased after transfection with sh-SNORA74B for 48 h in GBC-SD, SGC996, and NOZ cell lines (p<0.05). Furthermore, TUNEL assay showed that the mean number of TUNEL-positive cells was much larger in xenograft tumors formed by SNORA74B-silenced cells than those in control groups (P<0.05, Figure 4C and 4D), indicating that SNORA74B silencing promoted apoptosis of GBC-SD cells *in vivo*. In addition, the expression of a series of apoptosis-associated proteins in cell lysates and mitochondria were examined using western blotting. As shown in Figure 4E, the expressions of bax, cytosolic cytochrome C, and cleaved caspase-3 in GBC-SD cells were increased after treatment with sh-SNORA74B, while the expressions of bcl-2 and mitochondrial cytochrome C were decreased.

### SNORA74B silencing induced PHLPP expression and suppressed activation of AKT/mTOR pathway

**Figure 5:**
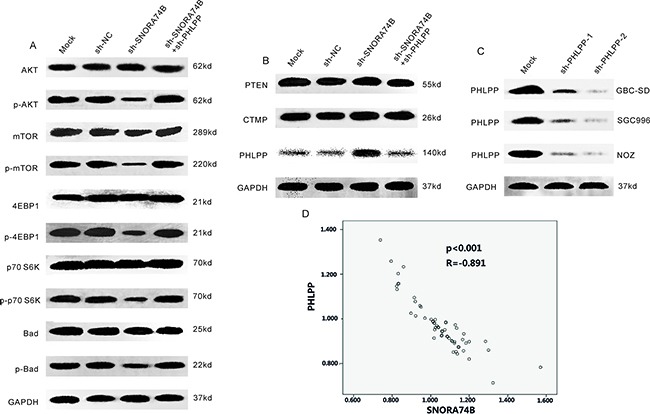
SNORA74B silencing induced PHLPP expression and suppressed activation of AKT/mTOR pathway **A**. Activation of AKT/mTOR signaling pathwaywas tested by western blotting after transfection, and the values of relative density of the bands normalized to GAPDH are shown. **B**. Expression of endogenous AKT inhibitors after transfection. **C**. PHLPP silencing in GBC-SD, SGC996, and NOZ cells by shRNAs. **D**. Correlation between SNORA74B and PHLPP expression in GBC tissues (n=59) (p<0.001, R=-0.891).

Overactivation of AKT/mTOR signaling pathway plays a prominent role in tumor initiation, progression, and prognosis. Phosphorylation of AKT and mTOR indicate activation of the AKT signaling pathway. In the present study, the protein level of members of the AKT signaling pathway, including AKT, mTOR, 4EBP1, p70S6K, and BAD as well as the endogenous inhibitors of the AKT pathway such as PTEN, PHLPP and CTMP were examined in GBC-SD cells after transfection using western blotting. As shown in Figure 5A, p-AKT, p-mTOR, p-4EBP1, p-p70S6K, and p-Bad protein levels were significantly decreased after transfection, indicating that AKT/mTOR pathway activation was suppressed. Figure 5B showed that PHLPP protein level was significantly increased, while the expression of PTEN and CTMP were not significantly changed after transfection. Furthermore, immunohistochemistry showed that SNORA74B gene silencing increased PHLPP protein levels in xenografts (Figure 2E), indicating that SNORA74B may regulate PHLPP expression both *in vitro* and *in vivo*. A negative correlation between the expression of SNORA74B and PHLPP in GBC cases was observed (Figure 5D). The above data suggest that SNORA74B regulates the expression of PHLPP in GBC. To determine whether the tumor suppressive effects of SNORA74B knockdown are mediated by PHLPP, we knocked down PHLPP by shRNA (Figure 5C) and examined the effects of SNORA74B silencing on the activation of members of AKT/mTOR signaling pathway, as well as on cell viability, cell cycle, and apoptosis. As shown in Figure 5A, once endogenous PHLPP is depleted, sh-SNORA74B no longer suppresses the activation of these members of the AKT/mTOR signaling pathway. In addition, proliferation inhibition, G1 arrest, and apoptosis induction caused by SNORA74B silencing are also abrogated (Figures 2C, 3, and 4).

## DISCUSSION

Over the past decades, the curtain has been gradually raised on the role of non-coding RNA in cancer biology especially miRNAs and lncRNAs. snoRNAs comprise a class of non-coding RNAs which has recently been linked to tumor development and progression. Since the initial discovery of C/D box snoRNAU50 involvement in B-cell lymphoma biology, several snoRNAs including SNHG20, SNORD78, and SNORD114-1 have been reported to act as oncogenes in colorectal cancer [7], lung cancer [8], and leukemia [9]. In the present study, we investigated the role of SNORA74B in GBC cell biology.

First of all, we found a significant increase in the expression of SNORA74B in tumor samples from 72 cases with GBC, and its levels were associated with local invasion, advanced AJCC tumor stage and Ki67 expression, but was negatively associated with PHLPP expression. Interestingly, we found levels of SNORA74B expression were positively correlated with CA19-9 levels. CA19-9 is a specific marker of gastrointestinal malignancy as these adhesion molecules may be secreted from cancer cells. It has been documented that serum CA 19-9 is a fairly good predictor for discriminating patients with gallbladder cancer from patients with gallstone and no cancer, and also gives a preoperative prediction of survival in GBC [10, 11]. If SNORA74B is detectable in blood, our result provides a possibility that SNORA74B combined with CA19-9 could serve as valuable diagnostic indicators for GBC. Future study should be carried out to clarify the link between SNORA74B and CA19-9. Kaplan-Meier analyses suggest that patients with higher SNORA74B expression have shorter OS and DFS after surgery. Univariate and multivariate analysis indicated that SNORA74B may serve as an independent predictor for GBC patients.

It has been documented that silencing of snoRNAs with oncogenic characteristics may inhibit the proliferation of cancer cells. For instance, silencing of SNHG16, which is up-regulated in colorectal cancer, inhibited proliferation, increased apoptosis, and reduced cell migration [12]. SNHG1 expression was significantly upregulated in both hepatocellular carcinoma and non-small cell lung cancer when compared with corresponding normal tissues and cells, and *in vitro* assays showed that knockdown of SNHG1 inhibited tumor cell proliferation [13, 14].

In the present study, by applying loss-of-function approaches, we identified that SNORA74B plays a role in cell proliferation, cell cycle progression, and induction of apoptosis *in vitro* and *in vivo*. To elucidate the potential molecular mechanisms by which SNORA74B silencing inhibits proliferation, induces G1 arrest, and promotes apoptosis, we examined the activation of AKT/mTOR pathway after siRNA knockdown. AKT/mTOR signaling pathway plays a key role in cell growth, survival, angiogenesis, and mobility [15]. Over-activation of AKT, which has been demonstrated to be oncogenic, is a frequent event in a variety of gastrointestinal tumors including colorectal cancer [16–19]. A number of studies have shown that the Akt/mTOR signaling pathway is over-activated in gallbladder carcinoma and deregulation of PI3K/Akt signaling is sufficient to transform gallbladder epithelial cells to cancer cells [20]. It has been documented that targeting AKT/mTOR signaling pathway may inhibit tumor growth and multiple inhibitors of this pathway have been developed and are being assessed in the laboratory and in clinical trials, including bevacizumab, gefitinib, and docetaxel [21, 22]. Besides exogenous agents, several endogenous inhibitors of AKT/mTOR such as phosphatase and tensin homolog (PTEN) [23], PHLPP [24], and carboxy-terminal modulator protein (CTMP) [25] have also been found recently.

In the present study, SNORA74B silencing suppressed Akt/mTOR activation, indicating that endogenous inhibitors of AKT/mTOR pathway may be activated. Therefore, we examined the expression of all these endogenous inhibitors and found PHLPP protein expression was significantly up-regulated after transfection with sh-SNORA74B. Moreover, PHLPP depletion abrogated the suppression of AKT/mTOR signaling, proliferation inhibition, G1 arrest, and apoptosis induction caused by SNORA74B silencing, indicating that SNORA74B-silencing-mediated antitumor activities depends on PHLPP function. PHLPP which is a tumor suppressor in several cancers may specifically dephosphorylate the hydrophobic motif of Akt, triggering apoptosis and suppressing tumor growth [26–28]. Importantly, it has been reported that PHLPP expression may be downregulated by miR-495 in GBC, while up-regulation of PHLPP may provide tumor suppressive effects through suppressing proliferation and inducing apoptosis [29]. Our data suggest that SNORA74B acts as a negative regulator of PHLPP, while the exact regulatory mechanism is not fully elucidated. Until now, little has been known about the molecular mechanism of snoRNAs function in cancers. Many non-coding RNAs, including snoRNAs, perform their functions through protein interactions. Zhao et al., found that SNHG5 exerted antitumor activities through binding to MTA2 oncogenic protein and preventing its translocation from the cytoplasm to the nucleus [30]. In addition, it has been documented in several tumor types that direct SNORD50A and SNORD50B RNA binding to K-Ras inhibits K-Ras oncogenic function [31]. Besides interacting with proteins, non-coding RNAs suppress protein-coding gene transcription by interacting and recruiting histone and chromatin remodeling proteins to target sites [32]. Both RNA-protein interaction and epigenetic suppression may be implicated in the regulation of PHLPP by SNORA74B. A further study on the interaction between SNORA74B and PHLPP should be carried out to enrich the existing knowledge on snoRNA functioning in cancer biology.

Taken together, our data suggest that SNORA74B plays an oncogenic role in GBC; silencing SNORA74B by shRNA may exert antitumor activities against GBC cell proliferation through inducing PHLPP and suppressing activation of AKT/mTOR signaling pathway.

## MATERIALS AND METHODS

### Microarray analysis

In our previous study, Human Gene 2.0 ST Array with total 33,340 probes (Affymetrix, CA, USA) was used to analyze the differentially expression of snoRNAs between GBC tissues and non-tumor tissues, including 1129 snoRNA probes, 9,289 IncRNA probes, 18,996 coding RNA probes and 1,426 miRNA probes [33]. The differentially expressed snoRNAs were identified and data are available via Gene Expression Omnibus (GEO) GSE62335.

### Patients and clinical samples

72 patients with GBC from Department of General Surgery, Xinhua Hospital, Shanghai Jiaotong University School of Medicine and Department of Gastrointestinal Surgery, The First Affiliated Hospital of Zhengzhou University between April 2010 and April 2014 were enrolled in this study. Fresh GBC tissues were collected immediately after surgery. The corresponding nontumor tissues were collected as negative control. The non-tumor tissue was cut from the adjacent tissue 5cm away from the tumor. Tissue samples was conserved in liquid nitrogen within 15 minutes after resection and part of the tissue was used for pathology analysis to distinguish tumor and non-tumor tissue. Histopathological diagnoses were reviewed by two histopathologists. None of the patients received preoperative chemotherapy or radiotherapy. The protocol of the study was approved by the Ethics Committee of Xinhua Hospital, Shanghai Jiaotong University School of Medicine and The First Affiliated Hospital of Zhengzhou University, and signed consent forms were obtained from patients recruited in this study. The patients were followed for 7 to 60 months (ended by the death of the patients) separately. Among these cases there were 47 deaths and 25 survivals.

### Cell culture

GBC-SD and SGC-996 were purchased from Cell Bank of the Chinese Academy of Science (Shanghai, China). NOZ and H69 was purchased from Japanese Collection of Research Bioresources Cell Bank (JCRB, Japan). SGC996 is established by Yang Y et al at 2003 and the cancer cells were isolated from the primary mastoid gallbladder carcinoma of a 61 year-old female patient [34]. GBC-SD is established by Liu B et al at 2000 and the cancer cells were isolated from the primary gallbladder carcinoma of a 61 year-old male patient [35]. NOZ is established by Homma S et al. The cancer cells were isolated from ascites of a patient of peritonitis carcinomatosa [36]. H69 is a human immortalized, nonmalignant cholangiocyte cell line [37]. The cell lines were cultured in Dulbecco's modified Eagle's medium (DMEM, Gibco BRL) supplemented with 10% fetal calf serum (FBS, HyClone) as well as 100 U/ml penicillin and 100 μg/ml streptomycin (Invitrogen). Cells were maintained in a humidified incubator at 37°C in the presence of 5% CO2. All cell lines were passaged for fewer than 6 months in our laboratory after resuscitation.

### RNA preparation, reverse transcription, and quantitative real-time PCR

Total RNA was extracted from tissues and cells using Trizol reagent (Invitrogen, Carlsbad, CA, USA) according to the manufacturer's protocols. cDNA was reverse transcribed from the extracted RNA using the Primer-Script™ one step RT-PCR kit (TaKaRa, Dalian, China). The cDNA template was amplified by quantitative real-time PCR (qRT-PCR) using the SYBR® PremixDimmer Eraser kit (TaKaRa, Dalian, China). qRT-PCR reactions were run on an ABI7500 apparatus (Applied Biosys- tems, Foster City, CA). The expression change of SNORA74B was calculated using a relative quantification (2-ΔΔCt method). Relative expression of SNORA74B was normalized to U6. Primer sequences of SNORA74B: F, 5′-CAGAACCGTTCCTGTGATGG-3′ and R, 5′- CAGCCAAAGTGAATGCTTAGC-3′.

### Cell transfection

To knock down SNORA74B expression, shRNAs that targeted SNORA74B (sh-SNORA74B-1 and sh-SNORA74B-2) and a scrambled negative control (sh-NC) were constructed by Sangon Biotech (Shanghai) Co., Ltd. Target sequences are listed as follows: sh-SNORA74B-1: GCUGGGAGAGGAAUGUCUUGU; sh-SNORA74B-2: GGGAGAGGAAUGUCUUGUCUU. The shRNAs that targeted PHLPP1 (sh-PHLPP1 and sh-PHLPP-2) were obtained from Santa Cruz Biotech. Inc. (Santa Cruz, CA, USA). Cells seeded in six-well plates were grown to confluency and transfected with Lenti-shRNAs using Lipofectamine 2000 (Invitrogen, Carlsbad, CA) according to the manufacturer's instructions. Transfected cells were harvested 48 hours after transfection and subjected to qRT-PCR or western blot analyses. To obtain stable transfectants, the medium was replaced with fresh complete medium supplemented with 10μg/ml puromycin after 48h of transfection. The selective medium was refreshed every 3-4 days until puromycin-resistant clones could be identified. SNORA74B expression levels in cell lines were examined by qRT-PCR and PHLPP expression levels were examined by western blotting.

### Immunohistochemistry

The expression of PHLPP and Ki67 proteins in GBC tissues were examined using immunohistochemistry. Frozen stored tissue specimens were embedded in optimal cutting temperature (OCT) compound and immunohistochemistry was performed as previously described [38]. The immunostaining intensity was evaluated using a 3-scale system (0, negative; 1, weak; 2, moderate; 3, strong). The percent positivity was also evaluated using a 3-scale system (0, <5%; 1, 5%-25%; 2, 25%-50%; 3, >50%). The overall quantitation for immunohistochemistry score was calculated by multiplying the score of staining intensity and score of percentage of positive cells. Ki67 and PHLPP expression levels were ranked as follows: - (score 0-1), + (score 2-3), ++ (score 4-6) and +++ (score>6). According to the scores of immunostaining, GBC patients were classified into two groups: low expression (− or +) and high expression (++ or +++).

### Measurement of cell proliferation

Cell viability was measured using CCK-8 assay according to the manufacturer's instructions. Briefly, transfected cells were trypsinized and seeded into 96-well plates (Corning, NY, USA) at a density of 5 × 10^3^ cells per well. After 1, 2, 3, 4, 5, 6, and 7 days culture, cells were incubated with CCK-8 solution for 1 h. The absorbance of each well was determined at 450 nm on a microplate reader (Bio-Rad, Hercules, CA, USA).

### Xenograft tumor model

Athymic BALB/c nude mice (age:4 weeks, weight: 20–25 g) obtained from the Shanghai Laboratory Animal Center of the Chinese Academy of Sciences (Shanghai, China) were used to establish the xenograft tumor models. Mice were housed under specific pathogen free (SPF) conditions. The experimental protocol was approved by the Ethics Committee of Xinhua Hospital, Shanghai Jiaotong University School of Medicine. GBC-SD cells were stably transfected with sh-SNORA74B or sh-NC. Approximately 1 × 10^7^ SNORA74B-silenced cells or control cells were suspended in a total volume of 200 μL of 1/1 (v/v) PBS/Matrigel (BD Biosciences, San Diego, CA, USA) and then subcutaneously injected into the flanks of the mice. 3-4 weeks post tumor cell injection, visible subcutaneous tumor volumes were measured every 5 days with calipers, and the tumor volumes in each group were calculated using the formula: volume = length × width^2^/2. 35 days later, the mice were sacrificed. The xenograft tumors were collected and weighed.

### Measurement of cell cycle

Cell cycle analysis was measured by flow cytometry. Transfected cells were seeded at a density of 1 × 10^6^ cells/well in six well plates. 48 h later, cells were fixed in ice-cold ethanol and incubated with propidium iodide (PI). The cell cycle distribution was analyzed using a flow cytometer (FACSCalibur, BD Biosciences, San Jose, CA, USA).

### Measurement of cell apoptosis

Cell apoptosis *in vitro* was measured using annexin V/ propidium iodide (PI) double staining assay according to the manufacturer's protocol (BD Pharmingen, San Diego, CA, USA). Briefly, 1×10^6^ transfected cells were plated in six-well plates and incubated for 48 h. Then, cells were collected and washed with cold PBS, centrifuged, resuspended in 400μL of binding buffer containing 5μL FITC conjugated annexin-V and 10μL PI and incubated for 15 mins at 4°C in the dark. A total of at least 10 000 events were analyzed by flow cytometry.

### TUNEL assay

Cell apoptosis in GBC-SD xenografts was assessed by Terminal deoxynucleotidyl transferase dUTP nick end labeling (TUNEL) staining using the one step TUNEL kit (Beyotime Institute of Biotechnology, Shanghai, China) following the manufacturer's protocol. Briefly, tissue sections were sequentially fixed in 4% paraformaldehyde, rinsed with PBS, and then permeabilized in 0.1% Triton X-100 for 2 min on ice, followed by the TUNEL assay for 1 h at 37°C. Afterward, tissue sections were counterstained with 4′,6-diamidino-2-phenylindole (DAPI). Cy3 (Cyanine 3)-labeled TUNEL-positive cells were viewed by fluorescence microscopy at an excitation of 488 nm and emission of 530 nm.

### Western blot analysis

Cells were transfected with shRNAs for 48 h and cytoplasmic and mitochondrial protein were extracted using Cell Mitochondria Isolation Kit (Beyotime, Shanghai, China). Protein samples were quantified using the bicinchoninic acid (BCA) assay (Beyotime, China). 30 μg of protein samples were separated by sodium dodecyl sulfate-polyacrylamide gel electrophoresis (SDS-PAGE) and subsequently transferred onto a nitrocellulose membrane (Millipore, Bedford, MA, USA). The membrane was blocked in 5% non-fat milk and then sequentially incubated with primary antibodies (1:5000-200) and the corresponding peroxidase-conjugated secondary antibodies (Santa Cruz Biotechnology). The detection of antibody binding was performed using the Supersignal chemiluminescence detection kit (Pierce, Rockford, IL, USA). Cox IV and GAPDH were used as the reference for mitochondria and cytosolic proteins.

### Statistical analysis

Results of all assays are expressed as mean ± standard deviation (m ± SD). All assays were performed independently in triplicate. All data were analyzed using SPSS version 19.0 software. The gene expression level of SNORA74B in tumors was compared with adjacent non-tumor tissues utilizing paired samples t-test. The relationship between SNORA74B expression and clinicopathological features were analyzed using chi-square. The difference in SNORA74B expression between cell lines, the expression changes after transfection, cell viability, xenografts volume and weight, cell cycle and cell apoptosis assays were analyzed using independent samples t-test. P<0.05 was considered statistically significant.

## SUPPLEMENTARY MATERIALS FIGURES AND TABLES


