# Supplementary material for: SNORA74B gene silencing inhibits gallbladder cancer cells by inducing PHLPP and suppressing Akt/mTOR signaling
Source: Oncotarget. 2017 Feb 13;8(12):19980–96. doi: 10.18632/oncotarget.15301 (PMC5386738; doi:10.18632/oncotarget.15301)
Supplement: Supplementary file 1 [file oncotarget-08-19980-s001.pdf]

SNORA74B gene silencing inhibits gallbladder cancer cells by inducing PHLPP and suppressing Akt/mTOR signaling

SUPPLEMENTARY FIGURE

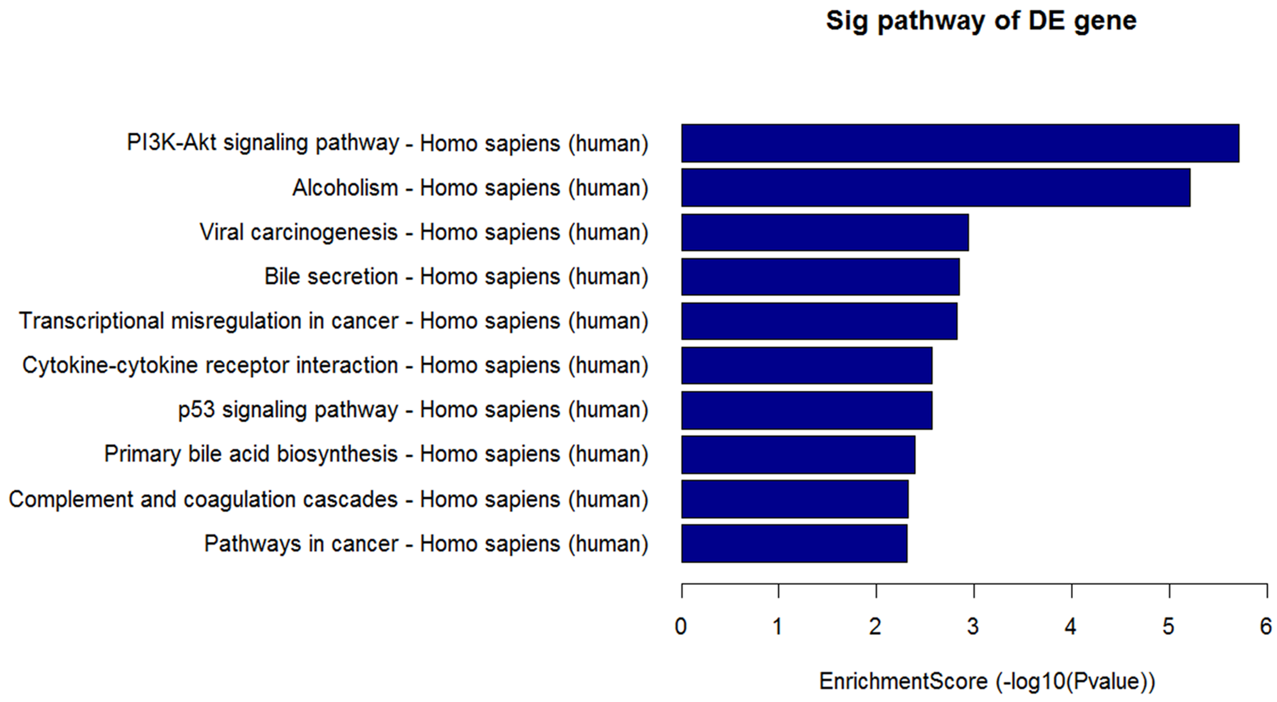

Supplementary Figure 1: Significant pathway of differentially expressed genes.
